# Impact of Gut Microbiota on the Peripheral Nervous System in Physiological, Regenerative and Pathological Conditions

**DOI:** 10.3390/ijms24098061

**Published:** 2023-04-29

**Authors:** Sonia Calabrò, Svenja Kankowski, Matilde Cescon, Giovanna Gambarotta, Stefania Raimondo, Kirsten Haastert-Talini, Giulia Ronchi

**Affiliations:** 1 Department of Molecular Medicine, University of Padova, Via Ugo Bassi 58/B, 35131 Padova, Italy; sonia.calabro@studenti.unipd.it (S.C.); matilde.cescon@unipd.it (M.C.); 2 Department of Biology, University of Padova, Viale G. Colombo 3, 35131 Padova, Italy; 3 Hannover Medical School, Institute of Neuroanatomy and Cell Biology, Carl-Neuberg-Str. 1, 30625 Hannover, Germany; kankowski.svenja@mh-hannover.de (S.K.); haastert-talini.kirsten@mh-hannover.de (K.H.-T.); 4 Department of Clinical and Biological Sciences & Neuroscience Institute Cavalieri Ottolenghi (NICO), University of Torino, Regione Gonzole 10, Orbassano, 10043 Torino, Italy; giovanna.gambarotta@unito.it (G.G.); stefania.raimondo@unito.it (S.R.); 5 Center for Systems Neuroscience Hannover (ZSN), Buenteweg 2, 30559 Hannover, Germany

**Keywords:** gut microbiota, peripheral nerve, nerve injury and regeneration, short chain fatty acid

## Abstract

It has been widely demonstrated that the gut microbiota is responsible for essential functions in human health and that its perturbation is implicated in the development and progression of a growing list of diseases. The number of studies evaluating how the gut microbiota interacts with and influences other organs and systems in the body and vice versa is constantly increasing and several ‘gut–organ axes’ have already been defined. Recently, the view on the link between the gut microbiota (GM) and the peripheral nervous system (PNS) has become broader by exceeding the fact that the PNS can serve as a systemic carrier of GM-derived metabolites and products to other organs. The PNS as the communication network between the central nervous system and the periphery of the body and internal organs can rather be affected itself by GM perturbation. In this review, we summarize the current knowledge about the impact of gut microbiota on the PNS, with regard to its somatic and autonomic divisions, in physiological, regenerative and pathological conditions.

## 1. Introduction

The collection of bacteria, viruses, fungi, and protozoans that colonize the gastrointestinal tract (GIT) is termed the ‘gut microbiota’ (GM). GM shares a mutually beneficial relationship with its host, and contributes to host tissue homeostasis through a series of physiological functions. These include digestion, vitamin synthesis, maintenance of the integrity of the mucosal barrier, protection against pathogens, immune system development and maturation, and central nervous system (CNS) modulation, thus making the GM a ‘vital-organ’ itself [1,2].

It has been estimated that the GIT is inhabited by more than 10^14^ microorganisms, which encompasses ∼10 times more bacterial cells than the number of cells in the human body and over 100 times the amount of genomic content (microbiome) as the human genome [3]. The GM is mainly dominated by the phyla *Firmicutes* and *Bacteroidetes* (up to 75% of all gut flora), and to a lesser extent by *Actinobacteria*, *Tenericutes*, *Proteobacteria* and *Verrumicrobia* [4,5].

At birth, the GIT is rapidly colonized by microorganisms; the microbial composition in early life is strongly influenced by the mode of delivery, the feeding method, the use of antibiotics and the maternal microbial composition. By around 2.5 years of age, the composition, diversity and functional capabilities of the human infant microbiota resemble those of human adult microbiota [3]. Various factors, such as geographical location, diet, exercise, use or abuse of antibiotics, age and diseases continue to shape and modify the GM composition during adulthood, to old age [3,6]. 

The GM transforms dietary nutrients and endogenous molecules into microbial-derived metabolites that can signal to peripheral organs and tissues in the body, allowing their communication through various mechanisms such as humoral, endocrine, immune and others, thus establishing complex ‘gut–organ axes’ [1]. The crosstalk between the GM and distal organs has therefore emerged as a research field of utmost interest and such interactions are being delineated piece by piece. In fact, in the last two decades, the number of studies showing the impact that the composition of the GM has on host physiology and metabolism and its interaction with various organs has grown substantially. The same is true for increasing evidence of an impact the body conditions itself, e.g., stress or drug treatment, have on the GM composition [7]. Alterations or imbalances in the amount, composition and diversity of the microbiota, a condition termed dysbiosis, has been linked to dysregulation of bodily functions and diseases such as inflammatory bowel disease, cardiovascular diseases, cancer, respiratory diseases, diabetes, brain disorders, chronic kidney diseases, liver diseases and others [8]. Therefore, identifying and controlling intestinal bacterial function with probiotics and prebiotics and/or modulating the release of bioactive factors from microbes represents an innovative approach for the treatment of multiple pathologies. 

It is now clear that the various components of the peripheral nervous system (PNS) serve not only as a carrier system between the GIT and other organs including the central nervous system (CNS), but are also influenced by the intestinal microbiota themselves. In particular, the importance of a healthy or well-balanced GM for the undisturbed development of the enteric nervous system has been widely demonstrated in recent years. To date, only few data exist in the literature on the possible interaction between the GM and the somatic part of the PNS, but recent findings support that there is indeed a direct interaction [9,10,11,12].

In this review, we will start describing the anatomy of the PNS and will then highlight currently available knowledge on how the different divisions of the PNS are influenced by the GM in physiological, regenerative and pathological conditions. 

## 2. Anatomy of the Peripheral Nervous System

**Figure 1:**
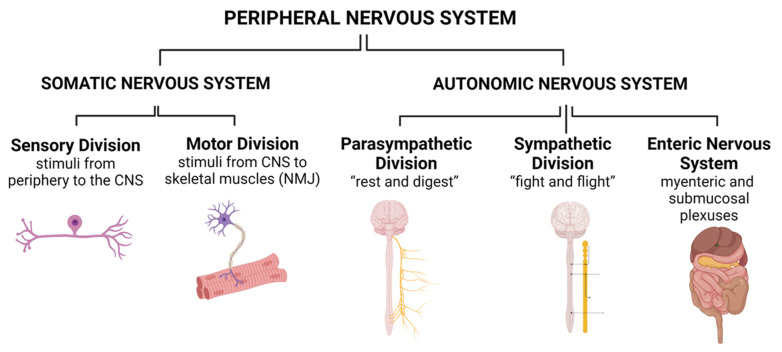
Organization of the Peripheral Nervous system. CNS: Central Nervous System. NMJ: Neuromuscular Junction. Created with BioRender.com.

In this section, we briefly recapitulate the anatomical organization of the PNS. The PNS can be divided into the somatic nervous system (SNS) and the autonomic nervous system (ANS). Both can be further subdivided into anatomically and functionally distinct subcategories (Figure 1).

The GM can communicate with its host organism via immunological, hormonal, and neuronal signaling for the regulation of digestion and metabolism [13]. Therefore, the mammalian organism needs to receive and process different kinds of information for maintaining a healthy condition and for interacting with its environment. The PNS with its neurons, axons, nerves, and plexuses, represents the “wiring” connection between the organisms’ outer and inner surfaces and the CNS (spinal cord and higher centers in the brain). Signals transmitted via peripheral nerves are processed towards the perception of internal and external stimuli (interoception or exteroception) followed by a motor response [13].

The axons of the peripheral nervous system therefore belong to different functional categories and either work as afferents, e.g., transmitting signals from an end organ to a neuron, or as efferents, e.g., transmitting signals in the opposite way. 

### 2.1. Somatic Nervous System (SNS)

The afferents in the SNS are represented by sensory axons transmitting tactile or painful stimuli towards the spinal cord or brainstem (sensory spinal or cranial nerves). The neurons responsible for this transmission have a pseudo-unipolar morphology and their cell bodies reside in the peripheral sensory dorsal root ganglia (DRG). The central sections of the sensory axons connect via the sensory, dorsal spinal roots to the spinal cord. Depending on the origin of the signal and the specific function of the DRG neuron, specific subclasses of DRG neurons form activating synapses with second-order neurons at different locations of the CNS. Second-order sensory neurons can be located in the dorsal horn of the spinal cord grey matter (e.g., for protopathic processing [14]), in the gracile or cuneate nucleus in the dorsal brainstem (e.g., for epicritic processing), or in the cerebellar cortex (e.g., for proprioceptive processing). Sensory stimuli processed via the SNS become conscious depending on the gating function of the diencephalic thalamus.

The efferents in the SNS are represented by motor axons originating from neurons with a multipolar morphology residing in the ventral horn of the spinal cord grey matter or motor brain stem nuclei. The respective motor axons connect via motor cranial or spinal nerves (e.g., via the motor, ventral spinal roots) with skeletal muscle fibers, where they form specialized synapses, the neuromuscular junctions (NMJs) [15], representing a specialized excitatory chemical synapse. At the NMJs, electrical impulses, transmitted via the efferent motor axons, are converted into muscle fiber action potentials. The conversion is achieved by the release of the neurotransmitter acetylcholine from presynaptic boutons formed at the distal end of a motor axon and high-density expression of acetylcholine receptors at postsynaptic invaginations of the sarcolemma [15]. Motor signals processed via the SNS are largely attributed to the voluntary control of skeletal muscles. 

### 2.2. Autonomic Nervous System (ANS)

The ANS, in contrast to the SNS, processes stimuli that do mainly remain unconscious and control smooth muscle fibers (e.g., in the vascular system or the GIT or the activity of the heart or lung). The ANS can be further subdivided into the parasympathetic, the sympathetic, and the enteric nervous system (ENS) [16]. 

The parasympathetic and sympathetic nervous systems contain afferents for processing sensory input signals and efferents for processing motor output signals that control the body homeostasis [13]. Signaling in the sympathetic system is transmitted via the sympathetic trunk and paravertebral and prevertebral ganglia to the peripheral organs [16]. The sympathetic signaling can be simplified as the one being responsible for the “fight and flight” conditions of the body [13]. The respective motor neurons are located in the intermedial nuclei of the lower cervical to the upper lumbar spinal cord segments. Signaling in the parasympathetic system is transmitted mainly via the vagus nerve with motor nuclei in the brainstem (medulla oblongata) and ganglia close to the target organs [16]. The system can be simplified as the one being responsible for the body “rest and digest” conditions [13]. 

The ENS is expanded as an intrinsic ganglia-rich neuronal network in the wall of the GIT and can be subdivided into the myenteric and the submucosal plexuses [17]. The myenteric plexus is located between the circular and longitudinal smooth muscle layers along the whole GIT from the esophagus to the anus. The presence of the submucosal plexus is restricted to the small and large intestines, where it is located beneath the mucosal layer of the gut [17]. The physical state of the intestine is detected by intrinsic primary afferent neurons in the submucosal and myenteric plexuses that can sense the interaction of chemicals with the mucosa as well as the distension caused by a food bolus [13,17]. The ENS forms a sensorimotor reflex circuit, independent of the CNS [18]. Enteric motor neurons can be classified as excitatory, inhibitory, secretomotor, vasomotor, and neurons for the enteroendocrine control [17]. Those located in the submucosal plexus regulate secretion and blood flow and those located in the myenteric plexus control gut motility [13]. Mainly located in the myenteric plexus, interneurons can also be found. They can be classified into orally or anally directed and form longitudinally expanded chains along the GIT [17]. The activity of the interneurons links the ascending and descending motor networks towards the autonomous functioning of the GIT [13].

We have now briefly reviewed the anatomy of the PNS. How GM can use the PNS in influencing developmental processes, in maintaining healthy conditions, or in the manifestation of diverse pathologies, is an active field of investigation [19]. The vagus nerve, however, represents an obvious interconnecting structure. Signaling molecules, produced in the GIT (e.g., microbiota-derived metabolites), could use the vagus nerve or also spinal nerves for reaching other parts of the body or the spinal cord, brainstem and brain. The vagus nerve is the main afferent pathway from the GIT and inner organs; it therefore provides an anatomical link between the internal organs and GIT to the brainstem with 80–90% afferent fibers and 10–20% efferent fibers. Sensory vagal inputs arrive in the nucleus of the solitary tract via the nodose ganglion. From there, fibers ramify to widespread areas of the CNS, including the cerebral cortex and medulla oblongata [20,21]. The vagus nerve is therefore able to sense the GM metabolites through its afferents, to transfer this gut information to the CNS where it is integrated into the central autonomic network for generating a well-adapted or an inappropriate response [22]. The integrity of vagal afferents is therefore critical for the communication of the GM via the PNS to the brain.

Molecular mechanisms that could mediate the communication between GM and the different PNS components have been recently reviewed elsewhere [13]. On the other hand, there is increasing evidence of a more direct impact of the GM on the proper development and function of the subdivisions of the PNS and their target tissues, which will be reviewed in the upcoming sections.

## 3. Effect of Gut Microbiota on the Somatic Peripheral Nervous System

The increasing knowledge that the composition of the GM can influence the regeneration of diverse tissues [23], including the liver [24], skin [25], and bones [26], triggered the evaluation of such impact also in the central and peripheral nervous systems. In the CNS, models of both traumatic brain and spinal cord injuries demonstrated that such insults are responsible for leaks in the gut barrier integrity, rapidly damaging intestinal mucosa. From there, toxins are systemically released in the plasma and further increase the intestinal permeability and malabsorption of nutrients. Moreover, the balance in gut bacteria populations is altered and even bacteria can be translocated to other organs [27,28,29]. As well, dysbiosis itself was recognized to influence the inflammatory response to injury and impairing functional recovery, therefore clarifying a bidirectional impact between the GM and the CNS in acute injury [28,30].

Evidence of a role for GM in contributing to peripheral nerve repair plasticity has emerged in the last years in the context of both the SNS and ENS. The following paragraphs will summarize updated knowledge from this field.

### 3.1. The Process of Somatic Nerve Injury and Regeneration

Long lasting effort in biomedical research was dedicated to the design of therapeutic approaches able to improve nerve regeneration after injury. Indeed, despite the peripheral neuron ability to regenerate, traumatic nerve injuries are conditions frequently leading to persistent motor and sensory impairment due to inefficient or delayed regeneration [31]. The regeneration process can be affected by many variables including the injury type, its extension, the requirement for surgery, the patient’s age, comorbidities and the lifestyle, including the diet [32,33,34]. Therefore, the research focusing on strategies promoting regeneration has still extensive room for application. In this context, a peerless contribution has come from the use of animal models of induced injury, that by unraveling cellular and systemically orchestrated mechanisms underlying the ability of peripheral nerves to regenerate, highlighted a number of relevant therapeutic targets [35]. A paradigm of timely defined events occurs upon nerve injury, starting from the interruption of the injured axonal fibers, leading to a disconnection between neuronal bodies and their distal terminals; these in turn undergo Wallerian degeneration, while a retrograde signaling from the injury site sets a communication with the neural nuclear core [36,37]. Schwann cells respond to the loss of axonal contact by triggering a re-programming that is essential in inducing cell proliferation, myelin debris removal, guidance of axon regrowth and remyelination [38,39]. This, together with immune cell recruitment, supports the establishment of a microenvironment that promotes the required regenerative process [40,41].

### 3.2. The Role of Gut Microbiota on Somatic Nerve Injury and Regeneration

The interest in the impact of GM on nerve injury and recovery has been growing very recently. In the last few years, studies emerged showing that GM modulation by antibiotics, prebiotics, or in pathological alterations such as dysbiosis can affect the recovery from peripheral nerve injury [42]. Liu et al. studied regeneration after corneal nerve injury induced by epithelial abrasion and demonstrated, for the first time, that the oral application of antibiotics results in a brake to the regeneration of the corneal branch of the trigeminal nerve (V cranial nerve) after injury. The application route for antibiotics had an impact on the GM composition, which was not affected after local application of antibiotics at the injury site via eye drop administration. Interestingly, the latter did not delay corneal nerve regeneration [9]. Indeed, mice orally treated with antibiotics displayed altered corneal gene expression pointing to a reduced neurogenesis, compared to control mice, as well as histological defects in corneal nerve regrowth. Of note, fecal microbiota transplantation (FMT) was able to rescue corneal gene expression and the density of regrown nerves; this was found to be mediated by CC chemokine receptor 2 (CCR2)-negative macrophages, a population of corneal macrophages, able to express neurotrophic cues and likely to be contributing to a regeneration promoting milieu [9]. When these cells were depleted by injection with anti-colony stimulating factor 1 receptor (CSF1R) antibody, regeneration was impaired, while CCR2-negative macrophages transfer from normal mice to antibiotics-treated ones normalized nerve healing. The expression of macrophage-related genes found to be downregulated upon oral antibiotic treatment was rescued not only with FMT, but also via probiotic administration, stressing the fact that GM composition has a direct impact on the immune cell state and distribution in peripheral districts [9]. Similarly, Rodenhouse et al. demonstrated that probiotics were able to counteract the delaying impact of oral antibiotic administration on motor functional recovery after traumatic sciatic nerve injury [10]. Therefore, in a scenario in which oral antibiotic treatment preceding nerve injury induced a reduction in the diversity of GM composition and consequently impaired nerve recovery, the administration of probiotics appeared to prevent regeneration deficits both in corneal and sciatic nerves. This contributes to the more general idea that dietary approaches targeting the balance in GM composition could promote peripheral nerve regeneration [9,10]. The probiotics applied so far in nerve regeneration, consisted in a commercial mixture, medical grade, of eight bacterial strains including Lactobacillaceae (*L. acidophilus*, *L. plantarum*, *L. casei*, and *L. bulgaricus*), Bifidobacteriaceae (*B. breve*, *B. longum*, and *B. infantis*) and *Streptococcus thermophilus*, referred to as VSL#3. This mixture was previously shown to exert beneficial effects on experimental models of irritable bowel syndrome, colitis and hepatic inflammation [43,44,45]. 

In line with the idea that a dietary approach can affect nerve regeneration by modulating systemic availability of the GM-derived products and metabolites, intermittent fasting was recently shown to enhance nerve regeneration and to sustain DRG neurite outgrowth upon sciatic nerve crush [11]. Despite the acknowledged key roles executed by Schwann cells and macrophages in the regeneration process, intermittent fasting, acting at a systemic level on cell metabolism, did not show any impact on the Schwann cell proliferation/dedifferentiation state, or on the recruitment of macrophages at the injury site [11]. Instead, this approach led to increased levels of detectable indole-3-propionate (IPA) in animal serum, paralleled by shifts in GM composition towards an enrichment in gram-positive bacteria and *Clostridiaceae* [11]. Indeed, oral antibiosis able to deplete gram-positive bacteria prevented the positive impact of intermittent fasting on nerve regeneration [11]. Fecal transplantation from intermittently fasting animals to control mice definitely demonstrated that the promoted nerve regeneration in intermittent fasted animals was due to the modulated GM. On the other hand, a direct impact of IPA on nerve recovery was confirmed via the oral administration of such metabolites. This, not only induced an improvement in nerve regeneration, but also a DRG-specific response in terms of the overexpression of genes involved in the recruitment of neutrophils, known to be among the first responders to injury and involved in Wallerian degeneration and axon regeneration also in the CNS [46,47]. The direct link between a higher presence of neutrophils within DRGs upon IPA treatment and axonal regeneration was evidenced by significantly impaired axonal regeneration upon neutrophil depletion [11]. If IPA treatment (or intermittent fasting) will become clinically relevant in the context of peripheral nerve injury has to be demonstrated. Some authors challenge the translational impact from rodent pre-clinical studies, because it has a long history of use as a neuroprotective reactive oxygen species scavenger in the brain in preclinical models, but was never reported to be used in patients, maybe due to differences between human and rodent PNS proteomes [48,49]. 

Short chain fatty acids (SCFAs) are monocarboxylic acids produced via bacterial fermentation of dietary fibers and resistant starch, and exert multiple beneficial effects on the host via multiple signaling mechanisms. Acetate, propionate and butyrate are the most abundant SCFAs (≥95%) and are present in an approximate molar ratio of 60:20:20 in the colon and stools [50]. The short-chain free fatty acid receptor FFAR is a G-protein coupled receptor (GPCR) activated by free fatty acids. In particular, FFA2 (GPR43) and FFA3 (GPR41) are activated by SCFAs. Free fatty acid receptor (FFAR) 3, but not FFAR2, has been recently shown to be expressed by Schwann cells and dorsal root ganglia [12]. Schwann cells and dorsal root ganglia exposed to oxidative stress and treated with propionate were reported to have better protection against oxidative damage by the downregulation of histone deacetylases (HDAC) 2 and upregulation of HDAC 8 expression with hyperacetylation of histone 3, resulting in the upregulation of the major antioxidative enzyme catalase; moreover, treatment with propionate also resulted in better DRGs axon outgrowth with increased expression of the growth associated protein 43 (gap-43) [12]. Taken together, these results demonstrated a neuroprotective and neuroregenerative effect of propionate in vitro.

### 3.3. The Influence of the GM on Motor Target Tissues of the Somatic Nervous System: The Gut–(Skeletal) Muscle Axis

The SNS is responsible for controlling voluntary skeletal muscle movements through neurotransmission at the level of the NMJ. Due to its ability to exploit insulin-mediated glucose uptake, fatty acid oxidation and breakdown of stored proteins for energy production, skeletal muscle is considered the major metabolic organ in the body. Moreover, it is highly plastic, being capable of responding to environmental stimuli, such as the training or nutritional states, by remodeling itself [51]. Interestingly, although no direct impact of the GM on somatic nerves has been reported to date, emerging scientific evidence supports the existence of a bidirectional communication between the intestinal microbiota and skeletal muscle, defined as the “gut–muscle axis”. In particular, within the past few years, an increasing number of animal studies reported a positive impact of a balanced GM on the maintenance of skeletal muscle mass and function. Compared to specific pathogen-free (SPF) mice, with usually undefined complex GM, germ-free (GF) mice and antibiotic-treated mice were found to display a reduction in muscle mass with signs of skeletal muscle atrophy [52,53,54,55]. Impaired grip strength and locomotor activity were also described in GF mice together with reduced serum levels of choline, the precursor of the neurotransmitter acetylcholine, and altered expression of NMJ-related genes, such as the ones encoding the different AChR subunits and the ones encoding rapsyn, low-density lipoprotein receptor-related protein 4 (Lrp4), and muscle-specific kinase (MuSK), key proteins involved in the formation and maintenance of NMJs [52]. Based on these findings, authors speculated that a defective nerve–muscle communication could occur under GM depletion [52]. In other studies, the ablation of the intestinal microbiota in mice via antibiotic treatment was similarly associated with reduced muscle endurance capacity and increased ex vivo muscle fatigue, but its impact on the NMJ structure and function was not investigated [53,54,56]. Interestingly, in both models, recolonization of the gut with a complex GM via either transplantation of fecal microbiota samples from specific pathogen-free animals or bacteria natural reseeding was effective in ameliorating overall muscle health, by increasing muscle mass and strength and, in some cases, also by improving endurance and fatigability [52,54]. A similar recovery was observed when GM-depleted mice were administered with a mixture of short chain fatty acids (SCFAs) or subjected to continuous acetate infusion, suggesting that GM-derived metabolites, and in particular SCFAs, could be important for promoting muscle anabolism and for regulating muscle energy expenditure [52,53]. 

**Figure 2:**
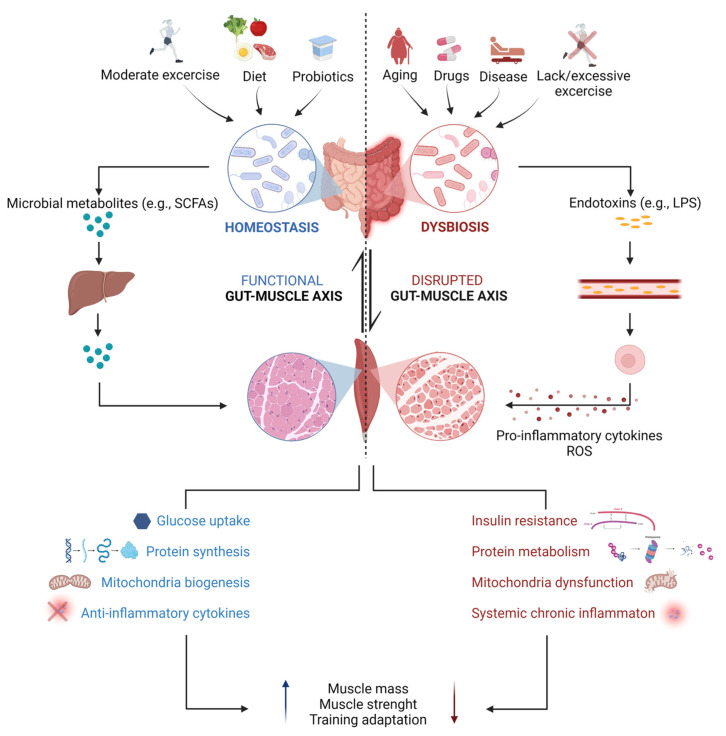
Schematic diagram of gut microbiota-mediated regulation of skeletal muscle under physiological and pathological conditions. The shaping of the gut microbiota composition is driven by both genetic and environmental factors. Regular moderate physical activity, consumption of dietary fibers and supplementation with prebiotics/probiotics promote an increase in the relative abundance of potentially beneficial bacteria, involved in the production of metabolites, among which short chain fatty acids (SCFAs). Following absorption by intestinal cells, SCFAs (mostly propionate and acetate, while butyrate is mainly used as a source of energy for colonocytes) reach the liver, where most of the propionate serves as a substrate for hepatic gluconeogenesis, while part of the acetate is destined to undergo hepatic lipid synthesis. Eventually, just small amounts of SCFAs (primarily acetate) reach the systemic circulation and directly interact with skeletal muscles [77]. Thus, both direct and indirect mechanisms concur with SCFA-mediated regulation of skeletal muscle metabolism, including modulation of glucose and energy metabolism, protein balance, mitochondrial biogenesis and regulation of inflammation, overall improving skeletal muscle mass and function. On the contrary, dysbiosis, a state that can be caused by aging, insufficient/excessive physical activity, drug consumption or disease state, is associated with increased intestinal permeability and gut leaking, which facilitates the passage of endotoxins (e.g., lipopolysaccharide, LPS) and other microbial products (e.g., indoxyl sulfate) into the peripheral circulation, from where these molecules trigger the production of pro-inflammatory cytokines and reactive oxygen species (ROS) by macrophages and promote insulin resistance, muscle proteolysis, and mitochondrial dysfunction in skeletal muscles. This results in skeletal muscle changes that may manifest as decreases in muscle mass and function, ultimately affecting muscle physical performance. Created with Biorender.com.

Besides its role in the modulation of skeletal muscle mass and functioning, the GM has also been demonstrated to influence skeletal muscle adaptation to exercise training. Indeed, in a recent work, gut dysbiosis was reported to prevent exercise-induced fiber-type shift and fiber-type-specific hypertrophy in mice [57]. On the other hand, several animal and human studies supported the idea that regular and moderate physical exercise positively modulates GM composition and activity by promoting microbial diversity and richness of beneficial bacterial strains involved in protein and carbohydrate metabolism as well as in SCFA production. Vice versa, training overload and sedentary behavior have been linked to dysbiosis with a decrease in anti-inflammatory bacteria and outgrowth of harmful opportunistic pathogens [58,59,60,61]. It is now clear that different mechanisms, both direct and indirect, concur for the regulation of skeletal muscle metabolism by the GM. An intact GM needs to be balanced with a healthy diet and an active lifestyle to increase glycogen storage capacity and mitochondrial function and to reduce oxidative and inflammatory stress, to promote muscle anabolism. Conversely, a dysbiotic microbiome in conjunction with an unbalanced diet and an inadequate exercise regimen are associated with insulin resistance, loss of mitochondria function, autophagy dysregulation, metabolic endotoxemia and consequent oxidative stress and systemic inflammation. Overall, these conditions lead to muscle proteolysis and therefore to a decline in muscle mass and function (Figure 2) [62,63]. Most of these observations, however, come from studies performed on young animals or humans, whereas less is known about this bidirectional interaction during aging. Physiological age-dependent changes in the GM composition occur in elderly people, where a reduction in bacterial diversity with an overgrowth of pathogens and a concomitant drop in anti-inflammatory species and butyrate-producers have been described [64]. Of note, a high inter-individual variability in the GM composition has been reported among older adults, also depending on their healthy status. Frail low-functioning older individuals, indeed, display a more altered GM and lower muscle strength compared to high-functioning volunteers of the same age, suggesting that a dysregulated gut–muscle axis may play a key role in the development and progression of age-related physical frailty and sarcopenia [65]. The mechanisms that have been proposed to mediate such an association comprise inflammation and anabolic resistance consequent to GM alterations, which could ultimately impact muscle size and function, as well as the modulation of appetite by microbial metabolites, which could on the other hand promote malnutrition [66,67]. Strategies aiming at modulating the intestinal microbiota have begun to be explored to counteract the loss of muscle mass and function in pathological conditions, including not only sarcopenia, but also cachexia and neuromuscular diseases, as well as to improve physical performance. Healthy diets (characterized by a high content of fibers and a low content of fat) and prebiotics/probiotics have been proven beneficial for skeletal muscle maintenance by several studies. A variety of bacterial species belonging mainly to the *Bifidobacterium* and *Lactobacillus* genera were found able to increase muscle mass, strength and endurance when they have been administered, sometimes in combination with training, to either adult or aged mice. The proposed mechanisms are restoration of mitochondrial dysfunction, attenuation of inflammation and increased glucose utilization [68,69,70,71,72,73]. Interestingly, similar results were reproduced in human studies [74,75,76]. Future studies aimed at deepening the understanding of the interplay between the GM and skeletal muscle in a more systemic view, e.g., considering also the PNS, could be of interest to clarify the mechanisms underlying GM regulation of host physiology. This type of studies could pave the way for the development of novel therapeutic approaches targeting the intestinal microbiota to fight muscle wasting and PNS abnormalities in the context of many physiological and pathological conditions.

## 4. Gut Microbiota and the Autonomic Nervous System

### 4.1. Link between the Gut Microbiota and the Sympathetic and Parasympathetic Nervous System

Is the regenerative process of the ANS also influenced by gut microbiota? Do injuries of the ANS affect the GM? Both these questions are still open since, to date, few publications are available regarding the correlation between the GM and ANS in its damaged/regeneration condition. 

Some researchers use an injury model of autonomic nerves for demonstrating how their lesions influence GM composition. For example, recently, Zhang et al. [78] demonstrated that a bilateral superior cervical ganglionectomy significantly caused abnormal GM composition, implicating a role for the sympathetic pathway in regulating the GM. Moreover, they demonstrated that this GM may participate in several biological pathways suggesting the important role of the cervical sympathetic ganglion in regulating the GM–brain axis, and further confirming that the sympathetic nervous system regulates the same.

Most of the articles use the denervation of the ANS for demonstrating the correlation between the GM and the brain. Liu et al. [21] emphasize the role of the vagus nerve in the GM to brain signaling as an integral component of a bi-directional neuroimmunoendocrine pathway. This way the vagus nerve does not act simply as a direct-line between the gut and brain; instead, the vagal signaling is required for an immune response that is a necessary and sufficient requirement for the effects of the bacteria on behavior. 

Surgical vagotomy has been used to investigate the physiological role of the vagus nerve in the gut–brain signaling and in certain diseases, including neurodegenerative diseases [79,80], depression-like behaviors [81] and others.

For example, two different register-based matched-cohort studies of vagotomized patients showed a reduced risk for subsequent Parkinson Disease among patients treated with truncal vagotomy [82,83], suggesting a critical role of the vagus nerve in the pathogenesis of the disease. Intriguingly, when the rat gut was injected with human brain lysate from patients with Parkinson Disease or with recombinant and pathological α-synuclein, α-synuclein has been demonstrated to be retrogradely transported into the brain via the vagus nerve, and this process was avoided via vagotomy [84,85].

Subdiaphragmatic vagotomy has also been shown to ameliorate demyelination and microglial activation in cuprizone-treated mice (an animal model for multiple sclerosis disease) compared to non-vagotomized animals [86]. It was also shown to block depression-like behaviors in Chrna7 knock-out mice (the gene coding for the α7 subtype of the nicotinic acetylcholine receptor), an animal model that demonstrates depression-like behaviors through abnormal composition of the GM and systemic inflammation [81], demonstrating the central role of the vagus nerve in the gut–microbiota–brain axis.

The indirect effect of ANS injury is also demonstrated by Guo et al. [87]. In fact, they demonstrated the beneficial effect of renal denervation on cardiac function and that this effect may be mediated by influencing intestinal bacteria, since it improves intestinal barrier function and ameliorates intestinal dysbiosis. On the other side, gut–microbiome aberrations and intestinal dysfunction are potential contributors to the development of heart failure [87].

Moreover, the renal denervation exerts beneficial effects on blood pressure control and perivascular fibrosis in rats exposed to chronic intermittent hypoxia, as demonstrated by Lu et al. [88]. Finally, Ma et al. [89], following a lesion in the splenic nerve, demonstrated that the GM might regulate microglial function in the brain via the gut–microbiota–brain axis.

### 4.2. Interactions between the Gut Microbiota and the Enteric Nervous System

In recent years, the interaction between the GM and the ENS has been extensively studied in both physiological and pathological conditions, as demonstrated by several recently published reviews that exhaustively describe this communication [13,18,90,91,92,93,94,95,96,97,98]. In this review, we will therefore provide only an overview of the impact of GM on the ENS structure and function, rather than describing in detail the complexity of the system. 

Abnormalities in the adult ENS morphology in GF animals have been demonstrated several years ago by the observation that the myenteric neurons of GF rats were hypertrophic compared to the controls [99]. Moreover, electrophysiological recordings showed decreased neuronal excitability in myenteric neurons of GF animals [100] with associated deficits in gut motility [101]. Alteration in terms of the loss of enteric neurons were also observed in systemically antibiotic (Abx)-treated mice [19]. GF mice display structural abnormalities of the ENS already during postnatal development (postnatal day 3) characterized by a decrease in nerve density and in the number of neurons per ganglion, and an increase in the proportion of myenteric nitrergic neurons with consequent functional alterations in terms of the frequency of amplitude of muscle contractions [102]. Going further back in the developmental stages of the ENS in utero, it has been demonstrated that exposing a pregnant sheep to *Ureaplasma parvum* infection results in the loss of enteric neurons in the ovine fetal ENS [103]. Interestingly, microbiota has also been demonstrated to influence enteric glial cell development [19,98,103]. GF mice showed lower numbers and density of enteric glial cells in the mucosa compared to control mice, but after recolonization with a normal GM, the number of enteric glial cells was restored [98]. Taken together, all these studies demonstrate that a healthy, or well balanced, GM is essential for the proper structural and functional development of both neurons and glial cells of the ENS. 

While the ENS is formed during fetal life (from embryonic day 8 to postnatal day 14 in mice and from week 4 in human embryos to the postnatal period) [104], plasticity persists in the postnatal period during which the GIT is colonized by bacteria. GF mice retain a higher degree of plasticity in the ENS with a higher proportion of the cells expressing nestin, a neuronal stem cell marker. Furthermore, the colonization of GF mice with conventional, complex GM induced the proliferation of enteric neurons with increased intestinal transit rates, through the release of serotonin (5-HT) and activation of 5-HT4 receptors [105]. Enteric neurogenesis has also been observed after microbiota recolonization in Abx-treated animals, with an increase in the number of enteric glia and neurons [19]. 

Several different mechanisms have been proposed to explain how the GM influences ENS functions. Different metabolites, by-products and molecules are secreted, modified, or stimulated by GM; among these, there are lipopolysaccharide (LPS) and Lipoteichoic Acid (LTA), tryptophan metabolites, serotonin, gamma-aminobutyric acid (GABA), bile acids, short chain fatty acids (SCFAs), cocaine-amphetamine regulated transcript (CART), substance P and calcitonin gene-regulated peptide (CGRP) [13].

Recent expression analysis indicated that enteric neurons and glia express receptors that can sense these metabolites, suggesting that they have the potential to respond directly to stimuli derived from the GM. For example, Toll-like receptors (TLRs) -3 and -7, which recognize viral RNA, and TLR4, which recognizes LPS (a membrane component of Gram-negative bacteria), are expressed in the myenteric and submucous plexuses (by both neurons and glial cells) as well as in the DRG neurons of the somatic PNS [106]. Mice lacking TLR4 displayed significant delay in gastrointestinal motility and reduced numbers of nitrergic ENS neurons, like GF mice. Furthermore, LPS activation of TLR4 in enteric neurons in vitro led to the activation of the transcription factor nuclear factor (NF)-κB and increased cell survival, suggesting that interactions between enteric neurons and microbes increases neuron survival and gastrointestinal motility in mice [107]. Moreover, TLR2, the receptor of LTA, is expressed by enteric neurons and it has been shown that the inhibition of TLR2 signaling suppresses neurogenesis whereas TLR2 agonists promote neurogenesis and restore myenteric neurons upon microbiota depletion [108].

Indole, an aromatic heterocyclic product of microbiota tryptophan metabolism, binds the aryl hydrocarbon receptor (AHR), a nuclear receptor controlling gene expression. Recently, AHR signaling in enteric neurons has been shown to be a key player in the maintenance of gut homeostasis. Mice lacking AHR, specifically in enteric neurons, showed reduced intestinal transit time, like GM-depleted mice. Intestinal motility is restored once AHR is expressed in enteric neurons of Abx-treated mice [109].

The most extensively studied gut microbe-derived metabolites in the ENS are SCFAs. FFA3 has been shown to be expressed by enteric neurons in both submucosal and myenteric ganglia [110,111]. A wide range of studies demonstrated the involvement of SCFAs as modulators of multiple colonic function, as well as of inflammatory and metabolic processes. SCFAs might be also involved in maintaining ENS homeostasis.

Butyrate, but not propionate or acetate, has been shown to significantly increase the proportion of cholinergic, but not nitrergic myenteric neurons both in vivo and in primary culture of ENS, in association with the ex vivo increase of colonic motility and contractile response; this neurochemical plasticity was reported to be mediated by Type 2 monocarboxylate transporter (MCT) and involves the Src kinase signaling pathway and the acetylation of histone H3 lysine 9 (H3K9) in enteric neurons [112]. Butyrate has also been shown to enhance both the cholinergic and nitrergic phenotypes of myenteric neurons and neuromuscular transmission postnatally [113]. Butyrate treatment also affects the postnatal development of enteric glial cells, since it has been reported to inhibit the proliferation of EGC in vitro and in vivo, without induction of cell death [114]. Propionate, on the other hand, has been shown to reduce the motility of the guinea pig colon [115].

A recent study showed that SCFA supplementation in Abx-treated mice rescues neuronal loss induced by Abx-treatment through enhancing neuronal survival and promoting enteric neurogenesis, but no effect on glial cells and only a minor effect on gastrointestinal function is reported [19].

The GM has been reported to also influence the extrinsic neurons of the gut [116,117]. Microbiota depletion resulted in elevated levels of gut-extrinsic sympathetic activity, as shown by the increased expression of the neuronal transcription factor c-Fos in the sympathetic coeliac-superior mesenteric ganglia. FMT from specific pathogen-free mice to GF mice restored the expression of c-Fos in the intestinal sympathetic ganglia, suggesting that microbiota can suppress the gut-extrinsic sympathetic neurons [116].

## 5. Microbiota in Pathological States of the PNS

Based on numerous observational findings collected in the last past decades in both pre-clinical and clinical settings, it is nowadays clear that an altered and less diverse GM is associated with a variety of pathological conditions. Interestingly, some recent studies highlighted how GM-related modulation of host physiology, especially through immunological and hormonal regulation, requires an interaction with the PNS. Moreover, both sympathetic and parasympathetic neurons are key regulators of gut functions and, consequently, of GM diversity and activity [118]. Given this close communication, it is easy to speculate that harmful alterations of this fascinating microbial community could lead to functional changes of the PNS, and vice versa, even in the context of non-peripheral pathologies.

### 5.1. Microbiota and Neuropathic Pain

Neuropathic pain is a sequela arising from micro- and macroscopic nerve lesions and diseases affecting the SNS and limiting patients’ quality of life. A large number of in vivo models has been established to investigate the molecular mechanisms underlying the development and chronification of neuropathic pain [14]. Studies performed in these models led to the hypothesis that alterations in GM may affect the development of neuropathic pain [119,120,121].

#### 5.1.1. Chemotherapy-Induced Neuropathic Pain

The GM was previously found to be a key player in the tumor growth-inhibiting effect promoted by different chemotherapeutic drugs in mice [122]. However, Shen et al. recently showed that it also mediates chemotherapy-induced neuropathic pain, an important dose-limiting side effect developed by more than 30% of patients undergoing continuous usage of chemotherapeutics, such as paclitaxel and oxaliplatin. Indeed, they found that the absence of a complete GM in both ABX-treated and GF-mice prevented oxaliplatin-induced mechanical hyperalgesia, a major symptomatic manifestation of neuropathic pain, and that this effect was abrogated when GM was restored with FMT [120]. The relevance of these pre-clinical findings is supported by a clinical study that revealed a change in the composition of the GM of patients who received oxaliplatin, compared to the one registered prior to chemotherapy [123]. Interestingly, several recent publications have demonstrated that microbiome composition is affected by host genetics in humans and that consequent variations in GM composition may impact the development of pathologies other than neuropathic pain [124,125]. In accordance with this finding, in their animal study, Ramakrishna et al. additionally observed different susceptibility to chemotherapy-induced peripheral neuropathies of different mouse strains, emphasizing the impact of the genome on the GM and thus on its associated pathologies [126].

Further data obtained from animal models suggest that the modulation of inflammation is involved in the protective effect of GM ablation on the development of neuropathic pain after chemotherapy. Shen et al. demonstrated that the DRG inflammatory response to the chemotherapeutic agent was dampened in the absence of a complete GM via the LPS-TLR4 pathway. In fact, following oxaliplatin treatment, the DRG from ABX-treated mice were found to display a decreased presence of infiltrated macrophages and inflammatory cytokines (IL-6 and TNF-α), compared to those of water-fed mice. The restoration of the inflammatory response upon exogenous LPS administration and the evidence that the specific ablation of TLR4 on hematopoietic cells was sufficient to phenocopy GM eradication support the role of GM-released LPS in promoting and intensifying the pro-inflammatory response of macrophages against oxaliplatin, while simultaneously leading to a hypersensitization of peripheral nociceptors [120]. The involvement of the LPS–TLR4 pathway in the insurgence of chemotherapy-induced pain is further confirmed in a study by Wardill et al. In their work, the authors show that irinotecan treatment in mice induced an increase in the permeability of the intestinal barrier, allowing LPS to be released from the GM and triggering neuropathic pain associated with spinal cord astrogliosis, both attenuated in treated TLR4^−/−^ mice [127]. Finally, another work proved that the administration of the probiotic formulation DSF (DeSimone formulation) to an in vitro model of DRG neurons was able to attenuate paclitaxel-induced neurotoxicity through the modulation of the inflammatory response [128], providing in this way, a rationale for the adoption of microbiota-targeting therapeutic strategies in the treatment of chemotherapy-induced peripheral neuropathy-associated neuropathic pain. 

#### 5.1.2. Diabetic Neuropathic Pain

Diseases of metabolic origin, such as obesity and type II diabetes, one of the most frequent comorbidities associated with obesity, are often accompanied by both autonomic and somatic alterations of the PNS as a result of dysregulated metabolic pathways, leading to the development of a painful peripheral neuropathy [129]. In this context, dysbiosis seems to play a pivotal role as a risk factor. Microbiome signatures from patients with diabetic neuropathic pain were recently found to differ from the ones of both healthy controls and patients with diabetes without diabetic neuropathic pain [130]. Interestingly, the observed enrichment in specific phyla and genera of bacteria, such as *Megasphaera* and *Parabacteroidetes*, positively correlated with insulin resistance, inflammation and dyslipidemia [130]. Of note, microbiota-modulating therapies, such as supplementation with probiotics or FMT, are effective in ameliorating insulin resistance [131,132]. A recent case report described remission from a painful neuropathy in an obese type II diabetic woman after FMT from a healthy donor [133]. In addition, FMT from lean to western diet-fed obese, insulin-resistant neuropathic mice was shown to prevent mechanical allodynia and thermal hyperalgesia and to decrease nerve fibers loss [134]. Pain relief was mainly achieved through FMT-mediated reduction of DRG neuronal hyperexcitability consequent to reduced Ryanodine receptor 2-dependent Ca^2+^ release from the endoplasmic reticulum. Furthermore, FMT was found to modulate PNS immune cells by promoting a shift in the polarization of DRG macrophages towards an anti-inflammatory phenotype and to increase circulating butyrate levels [134]. Even though such evidence points to microbiota-modulating interventions as promising tools to prevent and to alleviate peripheral neuropathies associated with obesity and type II diabetes, further studies will be necessary to elucidate the molecular mechanisms linking GM to pain and to discriminate between beneficial and detrimental bacterial strains.

#### 5.1.3. Trauma-Induced Neuropathic Pain

Injuries of the PNS are another major cause of neuropathic pain. Two independent groups of researchers draw a link between chronic constriction injury (CCI)-induced neuropathic pain and GM in rodents. Chen et al. reported an altered composition of intestinal microbiota in animals with CCI-induced neuropathic pain compared to sham controls [121]. This indicates that the condition of CCI-induced neuropathic pain somehow influences GM structure. An impact in the opposite direction, however, can also be postulated. Ding et al. demonstrated that changes in GM composition induced by ABX pre-treatment influenced the parameter values of CCI-induced neuropathic pain [135]. Indeed, following antibiotic treatment, animals presented with damped mechanical allodynia and thermal hyperalgesia. The translatability of these findings into human clinics, however, might prove difficult, because a large patient study, in contradiction to the pre-clinical observation of Ding et al. [135], demonstrated an increased incidence in neuropathic pain after antibiosis [136]. 

The mouse studies reported above [121,135] unfortunately do not provide information about pain management potentially applied to the operated animals and do not investigate whether the pain medication or the antibiotics might have directly influenced the observed development of neuropathic pain. This specific question was addressed in a study by Ma et al. demonstrating that FMT from healthy to neuropathic mice can reverse the amelioration of neuropathic pain arising from nerve injury, chemotherapy, and diabetes caused by antibiotic depletion of GM [137]. These observations are in accordance with the fact that in vivo treatment in mice with the phytosteroid diosgenin reversed oxaliplatin-induced peripheral neuropathy parameter values (e.g., mechanical withdrawal threshold and cold hyperalgesia). Again, treatment of the animals with FMT indicated that the pain ameliorating effect of diosgenin is GM-dependent [138]. 

To our knowledge, most research approaches investigating the influence of the microbiome on the development of neuropathic pain are based on the comparison of different mouse strains or of ABX-induced microbiome depletion models. Consequently, it has first to be considered that different genetic backgrounds might influence pain responsiveness per se and that therefore sensitivity to pain does not depend only on GM composition. Secondly, ABX may not alter the intestinal microbiota of different individual animals in the same way. Pane et al. questioned, in their systematic review, whether there is a correlation or a cause–effect relationship between the intestinal microbiome and neuropathic pain states [139]. 

For excluding confounders, future research should rather compare neuropathic pain development and related parameter values in long-term stable GF or gnotobiotic animals with that in animals from the same genetic background, but carrying a complex GM. The advantage could be obtained from the use of well-established and highly standardized models, such the axenic GF mice used by Shen et al. [120] or the oligo-mouse-microbiota (OMM^12^) model [140], containing only 12 defined bacterial strains. In our opinion, the comparison of standardized models of GM depletion would allow to increase the reproducibility of data and possibly to better elucidate whether there is a verifiable causality between GM composition and the prevalence in the development of neuropathic pain caused by different inducing conditions. 

### 5.2. Microbiota and Peripheral Implications in Autism Spectrum Disorder

Autism Spectrum Disorder is a heterogeneous and multifactorial neurodevelopmental disability that, in addition to core clinical manifestations (difficulties in social communication and interaction and repetitive behaviors), is often defined by the presence of GIT-related symptoms, whose severity positively correlates with the one of the pathology itself [141]. Many studies have documented that, compared to neurotypical people, autistic patients display gut dysbiosis with an enrichment of the *Clostridium* genus and an imbalance in the *Firmicutes*/*Bacteroidetes* ratio [142]. Another common pathogenic event in Autism Spectrum Disorder is ANS dysfunction, characterized by a hypoactivation of the parasympathetic branch with a concomitant hyperactivation also of the sympathetic branch [143]. Interestingly, in recent years, such autonomic imbalance has been hypothesized to be responsible for Autism Spectrum Disorder-associated dysbiosis through deregulation of the gut–brain axis at multiple levels [144]. First, parasympathetic stimulation is a known modulator of intestinal innate immunity, and deficiencies in its inputs may weaken the production of anti-microbial peptides from cells of the colonic mucosa, facilitating bacterial translocation and promoting both metabolic and oxidative stress. Second, ANS dysfunction could at the same time attenuate the enteric inflammatory response, thereby sustaining the already established dysbiosis state. Finally, functional alterations of ANS, which is responsible, together with ENS, for regulating water and electrolyte balance, could cause a perturbation of the intestinal osmotic equilibrium, stabilizing the GM in its altered form [144]. The instauration of a vicious cycle in which ANS dysfunction and gut dysbiosis mutually sustain each other could explain why therapeutic approaches targeting the microbiota have been applied so far to Autism Spectrum Disorder with only conflicting and transient results.

### 5.3. Gut Microbiota and the Sympathetic Nervous System in Hypertension

Gut dysbiosis has also been linked to hypertension [4,145,146,147]. Chronically enhanced sympathetic nervous system activity accompanied by a high release of noradrenaline and dampened parasympathetic activity are hallmarks of hypertension, thus suggesting a neurogenic component that contributes to the development of this disease [148]. Spontaneously hypertensive rats, as well as hypertensive patients, showed a significant decrease in microbial richness, diversity, and evenness, in addition to an increased *Firmicutes*/*Bacteroidetes* ratio (F/B ratio), which is a signature of gut dysbiosis [146]. On the other hand, under normotensive conditions, the reduction or the absence of GM (obtained with antibiotic treatment or with GF mice, respectively) does not significantly affect blood pressure [149,150].

A study in which reciprocal FMT from normotensive rats to spontaneously hypertensive rats showed that blood pressure was reduced in the hypertensive rats transplanted with feces from the normotensive rats and vice versa. Moreover, plasma noradrenaline levels were reduced in the first case and increased in the latter one, showing an impact of GM on the sympathetic nervous system activity. FMT from normotensive to hypertensive rats also induced a reduction of the inflammation in the hypothalamic paraventricular nucleus, a brain area for cardiovascular control, which also showed altered expression of receptors for SCFAs, suggesting a possible connection between GM metabolites and blood pressure control [151]. Indeed, spontaneously hypertensive rats were characterized by reduced acetate and butyrate-producing bacteria [146]. SCFAs have been reported to have anti-hypertensive properties, since their administration lowers blood pressure, and this effect is supposed to be mediated, at least in part, by G protein–coupled receptors, such as olfactory receptor 78 and G protein–coupled receptor 41 (GPR41) [152,153,154].

Moreover, intraperitoneal injection of acetate (the most abundant SCFAs in the plasma) results in a decrease of blood pressure and heart rate, and this last effect was blocked after treatment with a selective β-1 adrenergic receptor antagonist, demonstrating a role of sympathetic tone in the acetate-mediated response [155]. Authors hypothesized that SCFAs act upstream of the sympathetic nerve terminal to block the release of norepinephrine to β-1 receptors in the heart, inhibiting the canonical sympathetic signaling pathway that acts through G proteins. This effect is attenuated by the treatment with sympathomimetics. They also hypothesize that acetate acts directly on the heart in a different cell type by binding to a currently unknown G protein–coupled receptor [155].

Interestingly, the elevated splanchnic sympathetic nerve activity and mild gut pathology in juvenile prehypertensive rodents precede hypertension-related gut dysbiosis, suggesting that elevated gut sympathetic nerve activity modulates the gastrointestinal environment before the development of hypertension [156].

Dysfunctional autonomic nervous system in gut dysbiosis seems therefore to contribute to the development and maintenance of hypertension [4]. However, the cellular and molecular mechanisms involved in the gut–brain interconnection to control blood pressure are not yet fully addressed. Two main hypotheses have been proposed.

According to the first hypothesis, increased activity of the sympathetic nervous system in the gut in pre-hypertensive set-ups contributes to epithelial dysfunction and gut leakage, leading to intestinal dysbiosis. This results in an imbalance in the production of bacterial metabolites, including SCFAs, that is accompanied by an increased production and secretion of gastrointestinal hormones, such as 5HT, by the enterochromaffin cells as a consequence of an overstimulation of the adrenergic signaling. Local 5HT thus decreases the parasympathetic activity of intestinal vagal afferents via 5HT3 receptors (5HT3R), while 5HT released into circulation may affect vasculature and cause vasoconstriction. Furthermore, circulating 5HT can negatively modulate the transmission of feedback from the gut to the cardioregulatory regions of the brain. In the end, the resulting concomitant dysfunction of both the two branches of the ANS would, if not cause a hypertensive phenotype, at least promote its persistence in time [147].

Recently, a second mechanism has been proposed that involves miR-204, a microRNA that plays an important role in CNS function, and that is known to be affected by gut dysbiosis. According to the hypothesized mechanism, gut dysbiosis decreases miR-204 levels in the hypothalamus, which, in turns, increases brain derived neurotrophic factor (BDNF) and glutamate ionotropic receptor NMDA type subunit 2B (Grin2B) and consequently, sympathetic activity and noradrenaline release. Noradrenaline, by acting on ß1 receptors on the sinoatrial node and on α1 receptors on the vasculature, produces cardiac and vessel contraction, respectively. In parallel, parasympathetic nerve activity is decreased, leading to lower levels of acetylcholine and a reduction in cardiac contraction. Deregulation of both sympathetic and parasympathetic nerve activity, and the consequent deregulation of the levels of noradrenaline and acetylcholine, will induce abnormalities in the heart and vessels, ultimately resulting in hypertension [157].

### 5.4. Gut Microbiota and Autoimmune Disorders: A Focus on Myasthenia Gravis

Over the years, expanding scientific evidence pointed at disturbances of GM as a major risk factor for the development of both central and peripheral autoimmune diseases, among which multiple sclerosis [158,159] and rheumatoid arthritis [160]. In the wake of these findings, attention has recently been focused on the role of microbiota aberrancies in the pathogenesis of myasthenia gravis (MG), an acquired autoimmune disease of the NMJ characterized by impaired neuromuscular transmission that clinically manifests as focal or generalized muscle weakness and fatigability [161]. Gut dysbiosis and altered metabolite profiles have been described in patients with MG in several studies. Qiu et al. were the first to provide evidence that MG is accompanied by a dysbiotic GM, as expressed by a reduced overall phylogenetic diversity and altered structural composition. Specifically, at the phylum level, a depletion in *Firmicutes* and *Actinobacteria* with a concomitant enrichment in *Bacteroidetes* and *Proteobacteria* was described in MG subjects compared to healthy controls [162,163,164]. The consequent drop in the *Firmicutes*/*Bacteroidetes* ratio is consistent with what has been reported for other autoimmune diseases, such as irritable bowel syndrome and Crohn’s disease [165,166]. It is also reflective of a pro-inflammatory environment in which the altered GM weakens intestinal epithelial integrity, initiating an immune response that exacerbates the immunological imbalance already underlying the autoimmune disorder [162]. Consistently with these findings, an altered fecal metabolome with a decrease in the overall content of SCFAs, and in particular of propionate and butyrate, was observed in individuals with MG [162]. Since most of the differential metabolites detected in MG patients have a role in amino acid, microbial and nucleotide metabolism, it was suggested that the GM may contribute to the development of MG through the modulation of metabolic pathways and oxidative stress [164]. Interestingly, Zheng et al. managed to identify several altered microbial features that correlated with disease severity and were able to discriminate MG subjects from healthy controls with a 100% accuracy based solely on a combination of microbial and metabolic markers. Using a similar approach, Tan et al. were capable of distinguishing between two clinical subtypes of MG, ocular and generalized myasthenia gravis. Overall, these studies demonstrated the existence of a dysregulated GM composition and activity in patients with MG and proved the feasibility and the potential of fecal GM and metabolomic analysis as a novel non-invasive diagnostic tool for such pathology. 

The association between GM disturbances and the development of MG has been confirmed also in preclinical settings. In fact, GF-mice, transplanted with the microbiota of MG patients and subsequently immunized to induce MG, exhibited impaired motor activity, upregulation of inflammatory cytokines and dysregulated fecal metabolic pathways compared to mice transplanted with either a healthy GM or a combination of the two [164]. However, how these two events are correlated is still under investigation. Recently, it was hypothesized that alterations in the composition of GM could impinge on one of the main pathogenic events in MG, the presence of an immune disequilibrium in the T_h_17/T_reg_ (T helper 17/T regulatory cells) axis. Indeed, contrary to T_h_17 cells, whose levels are dramatically increased in MG patients, where they stimulate B cells to activate and to produce pathogenic autoantibodies [167,168], T_reg_ cells are significantly depleted in the blood of MG patients, contributing to the exacerbation of autoreactive immune responses [168]. Curiously, CD4 + CD25 + Foxp3 + T_reg_ are highly abundant in the colonic lamina propria, where they affect and can be affected by GM [169]. It has been proposed that a reduction in the proportion of *Clostridium* strains and, consequently, in the production of SCFAs, could profoundly impact the T cell development, favoring the conversion of naïve CD4+ T cells towards T_h17_ cells rather than T_reg_ cells [162,170]. Increased proportions of *Streptococcus* are likely to similarly restrain CD4 + CD25 + Foxp3 + T_reg_ differentiation through the inhibition of the activity of peroxisome proliferator-activated receptor γ (PPARγ) [162,170]. Moreover, increased levels of *Desulfovibrio* were suggested to contribute to MG pathogenesis by reducing butyrate levels and therefore by promoting an inflammation state [171]. Improving the current understanding of the types of microbial species and metabolites and of the mechanisms that underlie MG pathogenesis could help in the identification of novel potential therapeutic targets. Meanwhile, the validity of the rationale of using microbiota-targeting interventions to restore immune homeostasis through correction of the T_h_17/T_reg_ imbalance has been proven in a number of autoimmune diseases, including not only MG [172,173,174], but also irritable bowel syndrome [175] and autoimmune encephalomyelitis [176]. Nonetheless, to better apply these findings in clinical practice, further studies are needed to determine the types and the dosages of probiotics that could provide benefits to MG patients as well as the appropriate frequency and duration of the FMT approach.

### 5.5. Gut Microbiota and Myopathies

Apart from their primary effect on skeletal muscle structure and function, genetic myopathies are generally linked to alterations in muscle and lipid metabolism as well as to endocrine disturbances and chronic systemic low-grade inflammation. Even though myopathic patients often suffer from gastrointestinal dysfunction and altered gastrointestinal motility, a causal link between a dysregulated gut–muscle axis and the pathophysiology of musculoskeletal disorders has not been proven yet. Interestingly, two recent studies demonstrated for the first time a role for GM in shaping progression and severity of Duchenne muscular dystrophy (DMD), the most frequent and severe form of human myopathy. Indeed, both studies independently showed that mdx mice, the main DMD mouse model, are characterized by an altered GM composition with increased *Prevotellaceae* and significantly different metabolic profiles compared to age-matched wild type animals [177,178]. The enrichment of *Prevotella* in mdx mice was strongly correlated with aberrant immunity activation, gut inflammation and damaged muscular integrity [177]. Of note, microbiota depletion in mdx mice reduced chronic muscle inflammation and fibrosis deposition, but simultaneously aggravated the deregulation of glucose and lipid metabolism, enhancing fatty acid oxidation and promoting a shift in fiber-type toward a more oxidative phenotype. On the other hand, FMT with an eubiotic microbiota reduced muscle immunity and ameliorated muscle pathological features of mdx mice, including reduced myofiber size, increased fibrotic infiltrate and impaired muscle force [177]. Similarly, oral supplementation of sodium butyrate in late-stage dystrophic mdx mice restored muscle locomotion and strength to the same extent as deflazacort, a synthetic glucocorticoid widely employed in the standard care of DMD [178]. Kalkan et al. further demonstrated that a dysregulated GM participates in DMD pathology by exacerbating inflammation and autophagy impairment through the promotion of endocannabinoid system overactivity. Consistent with this finding, sodium butyrate exerted anti-inflammatory and pro-autophagic effects and reduced endocannabinoid system overactivity in mdx mice as well as in LPS-stimulated C2C12 myotubes and even in primary human myoblasts from DMD patients [178]. Even though still confined to preclinical settings, the results presented in these two studies open up the possibility to use dietary and microbiota modulating interventions to ameliorate or reverse symptoms in dystrophic patients and to slow down the progression of neuromuscular diseases. 

## 6. Conclusions

In this review, we aimed at gathering and presenting a concise overview of the currently available knowledge of the impact of GM composition on the different components of the PNS in physiological, regenerative, and pathological conditions. 

From this, it is obvious that the way to intensify the research in this field is open, given that promising potential for the modulation of the GM in the treatment of PNS pathologies still needs to be fully addressed. This opens the field to further experimental and clinical research, eventually leading to translational applications. 

A main limitation from a translational point of view is, however, represented by the low similarity between the different experimental models and the human being. There is a need for first securely proving causality and second defining the exact identity and optimal balance of microbe phyla or species that will be decisive for limiting specific pathologies. This is also envisioned by a new approach for specific depletion of well selected bacterial species in the human GM [7]. The strict use of highly standardized GF-models or gnotobiotic models appears more promising in this than the “simple” depletion and modification of the GM by systemic antibiosis [179].
